# The Relation of the Medical Profession

**Published:** 1921-08-06

**Authors:** 


### RATE-PROVIDED HOSPITALS.

### The Relation of the Medical Profession.

Considerable discussion took place at Newcastle when Sir George Newman, Chief Medical Officer of the Ministry of Health, speaking at a sectional meeting of the British Medical Association, outlined a plan for providing the medical service required by local authorities. He does not think that local authorities can meet their obligations to the ratepayers by a whole-time medical service, which in his view would sacrifice certain guarantees to success, *i.e.* the goodwill of the medical practitioners of the area, the co-operation of the voluntary institution, and the partnership in working. An adequate medical service, regular, economical, and available to all ratepayers on certain specified conditions and terms, should be possible by proper co-ordination between institutions and public and voluntary medical agencies in the area, by the selection of patients on grounds of urgency of physical need, and by the payment by or on behalf of the patient for part or all of the services needed. The following are the suggested means to provide such a service which Sir George commended to the consideration of the authorities of rate-provided institutions: (1) The association of representatives of medicine, by co-option or otherwise, with the Public Health Committee or other governing body; (2) a medical staff committee for the medical administration of the institution; (3) general practitioners of the area to be associated with the institution, on the staff or otherwise. Such an arrangement would secure their interest and support, provide facilities for post-graduate study, benefit the patients directly, and increase co-operation between the consulting staff of the institution and the practitioners of the neighbourhood; (4) payment of the medical staff, both consultant and practitioner, for their professional services, either by the authority or by the patient, on a fixed basis mutually agreed between the authority and the staff; the tenure of office to be fixed; such part-time staff to recognise the suzerainty of any whole-time medical officer; (5) a local medical advisory committee to be appointed by the medical profession in the district.

### Points in the Discussion.

Councillor David Adams, ex-Vice-Chairman of the Sanitary Committee of Newcastle Corporation, urged that a unified medical service is imperatively required. In his view a State medical and allied service must ultimately be evolved. The entire Poor-Law system, wasteful, costly, and repressive, ought to be abolished; and no aspect of Poor-Law administration is less satisfactory than that of medical service. He believes a statutory committee of the existing local authority would prove the most efficient instrument as health authority, on which could be co-opted a limited number of representatives of the medical and allied services. Dr. J. Middleton Martin, County Medical Officer of Health for Gloucester, gave an account of the scheme for the extension of medical services in that county which was adopted in July 1919.* Its practicability is confirmed by the fact that it is in operation; the estimate of cost for the first year, with twelve out-stations, was £5,000, and for the whole scheme £15,000, which is less than one shilling per head of the population. Colonel Nathan Raw, M.P., looks forward to the amendment of the National Insurance Acts, when he hopes the institutional and hospital treatment of all coming under the Act will be provided. He is opposed to a whole-time national medical service, believing it to be not conducive to the welfare of the people or of the profession. In his judgment all persons using a voluntary hospital should pay according to their means. When this is achieved, with a little help from the rates or from other sources, he anticipates the voluntary hospital will be able to carry on a municipal scheme combined with a voluntary scheme which should be quite sufficient to meet all demands. Mr. Roden Orde, House Governor and Secretary of the Newcastle Royal Victoria Infirmary, viewing the question from the point of view of [illegible] hospital administrator, deplores the lengthy waiting li[illegible], for which, as things are, there is no remedy. To the ordinary man there seems no reason why the unutilised hospital accommodation of many Poor-Law institutions should not be utilised; in fact, if the general public were to realise what that accommodation is, he is sure it would not authorise a single penny to be spent either in extending voluntary hospitals or building new ones until it had satisfied itself that there is something in the nature of things to render the utilisation of such Poor-Law accommodation impossible. He believes all the difficulties in the way can be overcome. The appropriate separation of buildings and affiliation with a voluntary hospital would cause a great alteration of opinion in regard to the alleged "pauper taint." He suggests that if approved societies are permitted to make grants to voluntary hospitals it would be equally proper for them to make grants to Poor-Law hospitals which are doing work supplementary to that of the general hospitals. Mr. Gladstone Walker (Newcastle) and Mr. Leach (Rochdale) were amongst those who spoke representing the Poor-Law authorities. The former submitted that though local authorities had had the power they had done practically nothing in the provision of hospital beds. The Guardians want to co-operate with the medical profession to enrich clinical fields, but they are held up in certain ways by the Ministry of Health. Mr. Leach believes that if the Health Ministry are going to arrest the movement for whole-time medical officers they are in for a fall; he suggested that on this question Sir George Newman was voicing his own opinion, and not that of the Ministry.

* See *The Hospital*, May 1, 1920, page 105.

